# Stimuli-Responsive Triblock Terpolymer Conversion into Multi-Stimuli-Responsive Micelles with Dynamic Covalent Bonds for Drug Delivery through a Quick and Controllable Post-Polymerization Reaction

**DOI:** 10.3390/pharmaceutics15010288

**Published:** 2023-01-14

**Authors:** Eva Hlavatovičová, Roberto Fernandez-Alvarez, Katarzyna Byś, Sami Kereïche, Tarun K. Mandal, Leonard Ionut Atanase, Miroslav Štěpánek, Mariusz Uchman

**Affiliations:** 1 Department of Physical and Macromolecular Chemistry, Charles University, Hlavova 2030, 12843 Prague 2, Czech Republic; 2 Institute of Biology and Medical Genetics, First Faculty of Medicine, Charles University and General University Hospital in Prague, Albertov 4, 12801 Prague, Czech Republic; 3 School of Chemical Sciences, Indian Association for the Cultivation of Science, Jadavpur, Kolkata 700032, India; 4 Faculty of Medical Dentistry, “Apollonia” University of Iasi, 700511 Iasi, Romania; 5 Academy of Romanian Scientists, 050045 Bucharest, Romania

**Keywords:** poly(4-vinyl pyridine), quaternization, poly(styrene)-*b*-poly(4-vinyl pyridine)-*b*-poly(ethylene oxide) terpolymer, micelles, alizarin, drug delivery systems

## Abstract

Stimuli-responsive copolymers are of great interest for targeted drug delivery. This study reports on a controllable post-polymerization quaternization with 2-bromomethyl-4-fluorophenylboronic acid of the poly(4-vinyl pyridine) (P4VP) block of a common poly(styrene)-*b*-poly(4-vinyl pyridine)-*b*-poly(ethylene oxide) (SVE) triblock terpolymer in order to achieve a selective responsivity to various diols. For this purpose, a reproducible method was established for P4VP block quaternization at a defined ratio, confirming the reaction yield by ^11^B, ^1^H NMR. Then, a reproducible self-assembly protocol is designed for preparing stable micelles from functionalized stimuli-responsive triblock terpolymers, which are characterized by light scattering and by cryogenic transmission electron microscopy. In addition, UV-Vis spectroscopy is used to monitor the boron-ester bonding and hydrolysis with alizarin as a model drug and to study encapsulation and release of this drug, induced by sensing with three geminal diols: fructose, galactose and ascorbic acid. The obtained results show that only the latter, with the vicinal diol group on sp^2^-hybridized carbons, was efficient for alizarin release. Therefore, the post-polymerization method for triblock terpolymer functionalization presented in this study allows for preparation of specific stimuli-responsive systems with a high potential for targeted drug delivery, especially for cancer treatment.

## 1. Introduction

Block copolymers in selective solvents undergo microphase separation, forming various types of nanoparticles. While in the case of diblock copolymers, the variety of formed structures is limited to spherical or cylindrical micelles and vesicles composed of solvophobic cores and solvophilic coronas, self-assembly of triblock terpolymers may lead to more elaborate nanoparticle structures with multiple domains, which offer a wider range of potential uses, such as nanocarriers in drug delivery. Triblock terpolymers can self-assemble into different types of nanoparticles based on the organization and properties of their building blocks [1,2,3]. The polymer structure and functionality strongly affect nanoparticle behavior under physiological conditions. Accordingly, modulating such properties allows for tailoring nanoparticles for specific purposes. The shape of the nanoparticles can be tailored by changing the order, length and solubility of their blocks [1,2,3,4,5,6,7,8], while their specificity and responsiveness to drug delivery can be enhanced by modulating the sensitivity to the external stimuli of its stimuli-responsive blocks and by introducing functional groups into the polymeric structure. Therefore, nanocarriers prepared from triblock terpolymers connecting blocks of various targeted specificity and responsiveness are highly promising as drug delivery systems [8,9,10,11,12,13].

A wide range of stimuli-responsive polymeric blocks have been studied for targeted drug delivery [14,15,16,17,18,19,20,21,22]. For instance, incorporating the pH-responsive poly(4-vinyl pyridine) (P4VP) block into a linear triblock terpolymer brings the possibility to change the structure of the micelles by varying the solubility of the block. P4VP can act as both a solvophobic polymer block in non-polar solvents and be solubilized or soluble in polar solvents, including dimethylformamide, tetrahydrofuran and aliphatic alcohols. P4VP solubility in water depends on the pH [23,24,25]. From neutral to basic conditions above pH 4.8, P4VP is water insoluble, but at a pH below 4.8, the 4-pyridyl groups of P4VP become protonated, rendering the polymers water soluble. Thus, placing a P4VP polymer block between a hydrophobic polymer block and a hydrophilic block, such as poly(styrene) or poly(ethylene oxide), results in triblock terpolymer micelles whose core radii vary as a function of the pH of the medium [26,27,28,29,30,31]. 

In addition to its intrinsic pH responsivity, P4VP is suitable for post-polymerization modifications through the quaternization of the nitrogen atom via multiple types of halogenated molecules [32,33,34,35]. The possibility of using a broad range of compounds as a quaternization agent opens many opportunities for tuning the system as desired.

**Scheme 1:**
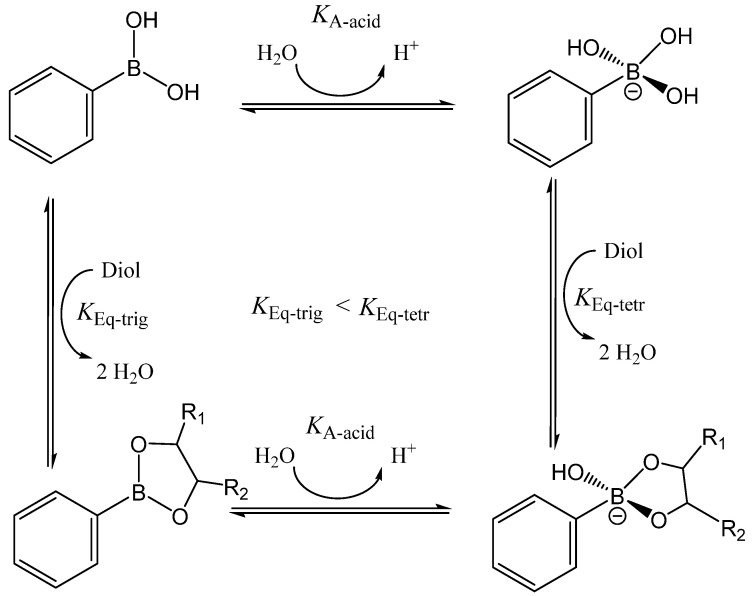
Phenylboronic acid-diol binding and ester hydrolysis with phenylboronic acid in trigonal (**top**) and tetragonal (**bottom**) conformations. *K*_A,acid_ and *K*_A,ester_ are the acid dissociation constants of phenylboronic acid and its ester, respectively, while *K*_Eq,trig_ and *K*_Eq,tetr_ are the equilibrium constants of the trigonal and tetragonal forms of PBA, respectively.

One of the options for such a modification is to use a derivative of phenylboronic acid (PBA) [36,37,38,39]. PBA is a stimulus-responsive molecule capable of binding vicinal diol-containing drugs, which has been used in several targeted delivery systems for its biocompatibility and reversible binding properties [37,38,39,40]. PBA can be found in two forms depending on the solution pH (Scheme 1). The neutral form is present in solutions with a pH below the p*K*_A_ of the phenylboronic acid (p*K*_A_ = 8.9) and gives the PBA properties of a mild Lewis acid. At pH > p*K*_A_, PBA turns into a negatively charged hydroxyboronate anion. Both forms can reversibly form esters with vicinal diols, but the esters formed by the anion are more stable [37,38,39,40]. Polymeric carriers with PBA moieties on side groups can thus be used for controlled drug release of vicinal diols if PBA derivatives with p*K*_A_ shifted to the physiological pH range (7.2–7.6) are used [37,38,39,40]. Under these conditions, they form stable esters with biomedically active vicinal diols, which can be released at a lower pH such as that found in tumor cells [41,42,43,44,45]. Thus, polymer-containing PBA molecules can be used not only as transporters for therapeutic agents but also as sensing molecules for various biomedical applications, due to their efficient encapsulation and release properties [41,42,43,44,45,46,47,48,49].

**Scheme 2:**
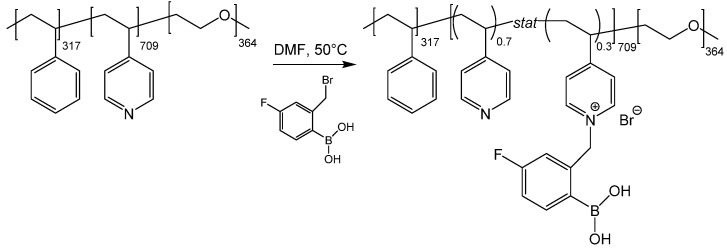
SVE quaternization with FPBA.

In this paper, we show that the P4VP block of a poly(styrene)-*b*-poly(4-vinyl pyridine)-*b*-poly(ethylene oxide) (SVE) triblock terpolymer can be reproducibly quaternized with 2-bromomethyl-4-fluorophenylboronic acid (FPBA), yielding an SVE-FPBA triblock terpolymer (Scheme 2) with PBA moieties on the side groups of the P4VP block. Using ascorbic acid, fructose, galactose and alizarin as model compounds, where alizarin allows for monitoring its binding to the copolymer with UV-Vis and fluorescence spectroscopies, it is demonstrated that SVE-FPBA micelles in aqueous solutions exhibit a strong ability to bind vicinal diols, and this post-polymerization reaction thus has the potential to be used for preparation of micelles with the ability to transport and release vicinal diol-containing drugs.

## 2. Materials and Methods

### 2.1. Materials

Poly(styrene)-*b*-poly(4-vinyl pyridine)-*b*-poly(ethylene oxide) (SVE) and poly(4-vinyl pyridine) (P4VP) were purchased from Polymer Source, Inc. (Dorval, QC, Canada). The molar masses of the individual polymer blocks *M*_n_ of SVE were 33, 83 and 16 kg/mol for polystyrene, poly(4-vinylpyridine) and poly(ethylene oxide), respectively, while the *M*_n_ of P4VP was 6 kg/mol. The dispersities *Đ* were 1.15 for SVE and 1.2 for P4VP. The 2-bromomethyl-4-fluorophenylboronic acid, FPBA (95%) and D2O (99.8 atom %D) were from Combi-Blocks (San Diego, CA, USA) and from ARMER Chemicals, respectively. Other chemicals were obtained from Sigma-Aldrich (St. Louis, MO, USA). All chemicals were used as received.

### 2.2. Quaternization of the SVE Triblock Terpolymer with FPBA

**Figure 1:**
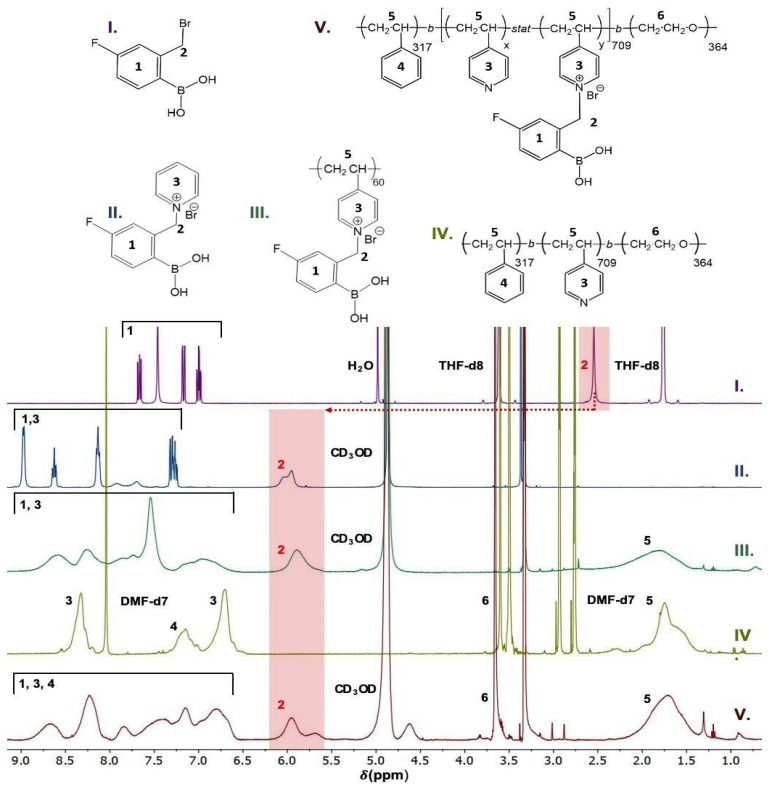
^1^H NMR spectra of (I) FPBA, (II) Pyr-FPBA, (III) P4VP-FPBA, (IV) SVE and (V) SVE-FPBA. The shift of the methylene linker after quaternization is highlighted in red.

The quaternization reaction occurred as follows (Scheme 2). First, 200 mg of SVE was dissolved in 12 mL of DMF in a round-bottomed flask under stirring at 60 °C. Then, 132 mg of FPBA (0.5 molar eq. per 4VP unit) was dissolved in 3 mL of DMF and added dropwise into the SVE solution, and the reaction mixture was kept for 5 days at 60 °C under stirring. The color of the reaction mixture changed from light yellow to dark green. The sample was then extensively dialyzed against water to replace the DMF. The dialysis bath was exchanged three times—twice after 4 h and once after 12 h—to completely remove the DMF. The dialyzed solution of SVE-FPBA was subsequently lyophilized, yielding a light green voluminous solid. The product of the reaction was analyzed by NMR spectroscopy (Figure 1). The degree of quaternization of SVE-FPBA was 30%.

SVE-FPBA: ^1^H NMR (400 MHz, CD_3_OD) δ (ppm) for 8.87–8.49 (s), 8.43–8.08 (s), 7.94–7.72 (s), 7.70–6.58 (broad triplet), 6.10–5.55 (d), 6.11–5.84 (d), 3.71–3.51 (s) and 2.17–1.23 (broad multiplet). ^11^B [^1^H] NMR (400 MHz, CD_3_OD) δ (ppm) for 18.54 (s).

### 2.3. Synthesis of N-[(5-fluoro-2-boronatophenyl)methyl]pyridinium Bromide

The product of the quaternization reaction of pyridine with FPBA, N-[(5-fluoro-2-boronatophenyl)methyl]pyridinium bromide (Pyr-FPBA), was synthesized as follows. First, 500 mg FPBA was dissolved in a round-bottomed flask with a magnetic stir bar in dried THF (5 mL) before subsequently adding pyridine (0.188 mL, 0.982 g/mL; 2.33 mmol). The reaction mixture was stirred at 60 °C for 2 days under an argon atmosphere. The liquid phase was decanted from the partly precipitated product (Pyr-FPBA) and poured into cold diethyl ether in order to precipitate the remaining product. Both precipitates were dissolved in methanol to remove reagents, and the methanol was then evaporated on a rotary evaporator. In total, 0.555 mg (81% yield) of product was isolated as a white powder and analyzed by NMR spectroscopy.

Pyr-FPBA: ^1^H NMR (400 MHz, CD_3_OD) δ (ppm) for 9.03–8.91 (d), 8.69–8.56 (t), 8.21–8.07 (t), 7.70–7.60 (m), 7.55 (m) and 6.11–5.84 (d).

### 2.4. Micelles Preparation

The SVE-FPBA micelles were prepared as follows. First, 10 mg SVE-FPBA dissolved in 1 mL of MeOH was added dropwise into 3 mL of water or 10 mM HCl. The mixtures were then extensively dialyzed against water or 10 mM HCl. The final concentration and pH of the samples after dialysis were 2.3 mg/mL and 7.2, respectively, for the solution in water (further referred to as N-SVE-FPBA) and 2.3 mg/mL and 5.1, respectively, for the solution in 10 mM HCl (further referred to as A-SVE-FPBA). To encapsulate alizarin (AL) into the SVE-FPBA micelles, 10 mg of SVE-FPBA in 1 mL of MeOH (1 mL) was mixed with 2.14 mg of AL dissolved in 1 mL of MeOH at 60 °C under stirring, yielding a deep pink transparent solution. The mixture was then added dropwise into 2 mL of water and then dialyzed extensively against water. The final concentration of SVE-FPBA in the aqueous solution was 1.85 mg/mL.

### 2.5. Methods

#### 2.5.1. ^1^H and ^11^B NMR Spectroscopy

The ^1^H and ^11^B NMR spectra were recorded at 25 °C with a Varian Unity Inova 400 MHz spectrometer in quartz cuvettes using THF-d8 and DMF-d7 as solvents for FPBA and SVE, respectively, and CD_3_OD as a solvent for Pyr-FPBA (16.7 g/L) and SVE-FPBA (10 g/L). The solvent peaks were taken as references in all measurements. 

#### 2.5.2. Static and Dynamic Light Scattering

The light scattering measurements were performed on an ALV photometer (ALV, Germany) consisting of a CGS-3 automatic goniometer, a 7004 multi-tau multibit autocorrelator, two high-QE APD pseudo cross-correlation detectors and a 100 mW, 660 nm diode-pumped solid state laser. Nanoparticle solutions with a polymer concentration *c* = 0.7 g/L were prepared at least a day before the measurements, which were carried out at 25 °C and at scattering angles *θ* ranging from 60° to 150°, corresponding to the scattering vector magnitudes *q* from 12.7 to 24.4 μm^−1^. The gyration radii and molar masses of the NPs were obtained by the Zimm method [50], neglecting the virial term (*A*_2_*c* = 0) and using the refractive index increment of P4VP-FPBA (0.207 mL/g). This value was calculated, considering the composition of the copolymer, as a mass average of the refractive index increments for PS (0.257 mL/g), P4VP (0.254 mL/g), PEO (0.130 mL/g) and P4VP quaternized with FPBA (0.165 mL/g). The latter value was obtained in our laboratory, while the other values were taken from the literature [51]. The hydrodynamic radii *R*_H_ were calculated using the Stokes–Einstein equation from the diffusion coefficients, which were obtained by extrapolating the Г_1_(*q*)/*q*^2^ values to *q* = 0, where Г_1_(*q*) is the first-order cumulant of the second-order cumulant fit [50] of the time-averaged field autocorrelation function. The size dispersity of the nanoparticles was expressed by the width of the distribution function of diffusion coefficients *σ* = Г_2_(*q*)/Г_1_^2^(*q*), where Г_1_(*q*) and Г_2_(*q*) are the the first- and the second-order cumulants at *q* = 17.9 μm^−1^ (*θ* = 90°), respectively.

#### 2.5.3. Zeta Potential Measurements

*The ζ* potential measurements were performed on a Nano-ZS Zetasizer (Malvern Instruments, Malvern, UK). The *ζ* potential values were calculated from the electrophoretic mobilities (average of three subsequent measurements, each consisting of 15 runs) using the Henry equation in the Smoluchowski approximation *μ* = ε*ζ*/*η*, where *μ* is the electrophoretic mobility, *η* is the solvent viscosity and *ε* is the dielectric constant of the solvent. The concentration of samples A and N SVE-FPBA was 1.32 mg/mL.

#### 2.5.4. Cryo-TEM

Cryo-TEM measurements were obtained with a Tecnai Sphera G20 microscope (FEI, Hillsboro, OR, USA). Images were recorded at a 120 kV accelerating voltage and under magnifications ranging from 5000× *g* to 14,500× *g* using a Gatan UltraScan 1000 slow scan CCD camera. Lacey-carbon grids (LC200-CuC, Electron Microscopy Science, Hatfield, PA, USA) hydrophilized by a glow discharge for 40 s with a 5 mA current were used for sample preparation, and 3 μL of the micelle solution was applied to the grid, which was blotted with filter paper and immediately plunged into liquid ethane held at –183 °C. The sample was then transferred without rewarming into a Gatan cryo holder. The size distribution of the micelles was assessed by analyzing a population of 50 micelles for both A-SVE-FPBA and N-SVE-FPBA using manual diameter measurements in ImageJ software.

#### 2.5.5. UV-VIS Spectroscopy

The absorbance measurements were performed at room temperature on a Shimadzu UV-VIS spectrophotometer. The samples were measured in 1 cm quartz cuvettes at a speed rate (0.5 nm/s) in the wavelength range from 300 to 700 nm.

## 3. Results and Discussion

### 3.1. Quaternization of Poly(styrene)-b-poly(4-vinyl pyridine)-b-poly(ethylene oxide)

**Figure 2:**
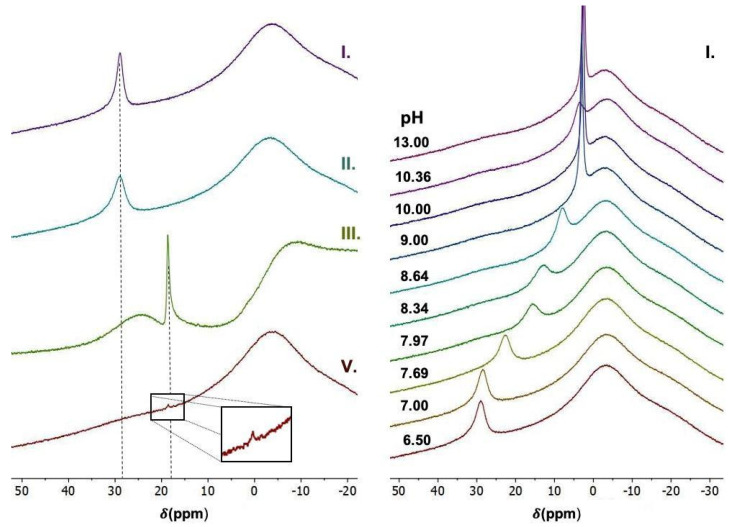
**Left**: ^11^B NMR spectra of (I) FPBA, (II) Pyr-FPBA, (III) P4VP-FPBA and (V) SVE-FPBA. **Right**: ^11^B NMR spectra of FPBA at various pH levels.

First, ^11^B NMR (Figure 2) was used to confirm the presence of boron units in the polymeric chain and to determine an approximate p*K*_A_ of the FPBA. The modification with phenylboronic acid was confirmed by the shift in the typical boron ester signal from 28 ppm to ~20 ppm. Furthermore, the pH effect on the boron ester signal shift in the monomeric analogue Pyr-FPBA allowed us to determine the p*K* of FPBA attached to the pyridinium group. The p*K*_A_ values were determined by fitting the experimentally obtained dependence of the boron ester signal chemical shift (PPM) on the pH level with the equation

(1) $$\delta =\frac{\delta_{\min}+\delta_{\max}10{\;}^{-pK_{A}-\mathrm{pH}}}{1+10{\;}^{-pK_{A}-\mathrm{pH}}\;}$$

where *δ*_min_ and *δ*_max_ are chemical shifts in the low and high pH limit, respectively. The pH of the solution was calculated from the pD using the relation pH = pD + 0.4 [52]. The determined p*K*_A_ of the monomeric analogue was p*K*_A_ ≈ 7.75. However, we must consider a possible deviation of the p*K*_A_ through phenylboronic units bound to the triblock terpolymer system. As such, we could use this value only as an estimate. Nevertheless, this p*K*_A_ estimate was necessary for the fluorophore encapsulation and release.

### 3.2. pH-Dependent Self-Assembly and Static and Dynamic Light Scattering 

**Figure 3:**
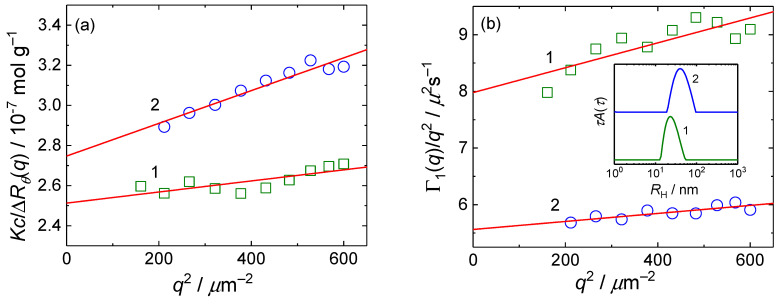
(**a**) Zimm plot and (**b**) dynamic Zimm plot for aqueous solutions of N-SVE-FPBA (curve 1) and A-SVE-FPBA (curve 2) micelles. Insert in (**b**) shows CONTIN distributions of hydrodynamic radii.

The self-assembly of SVE-FPBA micelles under two different conditions (A-SVE-FPBA and N-SVE-FPBA, described in the Materials and Methods section) was analyzed by static and dynamic light scattering and by electrophoretic light scattering in order to determine the averaged molecular weight of the micelles (*M*_W_), the radius of gyration (*R*_G_), the hydrodynamic radius (*R*_H_) and the *ζ* potential (Figure 3 and Table 1).

The A-SVE-FPBA micelles had lower *M*_W_ values than the N-SVE-FPBA micelles because of the higher solubility of P4VP-FPBA at lower pH levels, which decreased the aggregation number of SVE-FPBA in the aqueous solutions. On the other hand, the A-SVE-FPBA micelles had larger *R*_G_ and *R*_H_ values because of the expansion of the protonated P4VP-FPBA shell at a lower pH because of electrostatic repulsion. The effect of the pH on poly(2-vinyl pyridine) with similar properties has been described previously [53,54]. The *R*_G_/*R*_H_ ratios were lower than the theoretical value for the homogeneous sphere *ρ*_sph_ = (3/5)^1/2^ [49], because the weakly scattering PEO corona contributed only a little to the *R*_G_ while it was still affecting the hydrodynamics of the micelle and thus contributing to the *R*_H_.

Both the A-SVE-FPBA and N-SVE-FPBA samples had a high *ζ* potential of +40 mV, indicating good electrostatic stabilization at both pH 5.10 and 7.20.

It is worth mentioning that neither the *R*_G_ nor *R*_H_ of the A-SVE-FPBA micelles increased further, and the *ζ* potential even decreased to +20 mV when the pH of the solution was adjusted to 2.5 by adding HCl. This behavior can be explained by the increased screening of the electrostatic charge of P4VP-FPBA blocks at an increased ionic strength for the solution, which compensated for the increased degree of protonation of the P4VP-FPBA blocks.

### 3.3. Micelles Imaging by Cryo-TEM

**Figure 4:**
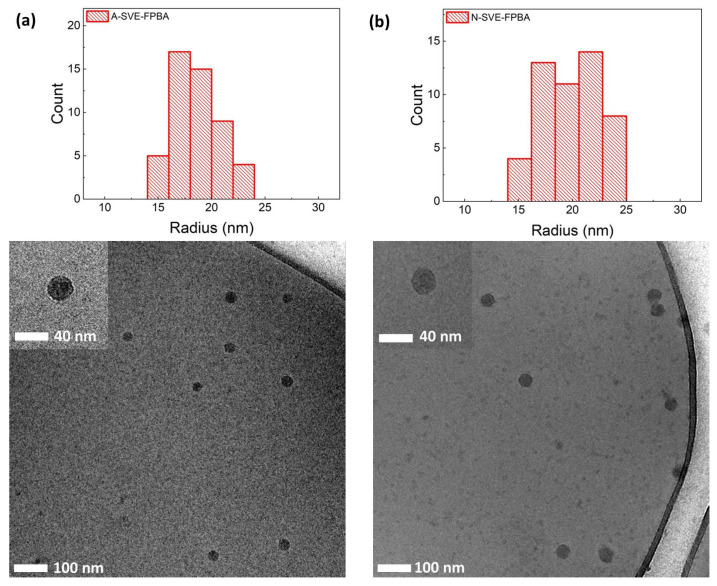
Cryo-TEM images of (**a**) A-SVE-FPBA (pH 5.10) and (**b**) N-SVE-FPBA (pH 7.20) micelles.

Cryo-TEM images from A- and N-SVE-FPBA are shown in Figure 4, together with histograms of the particle radii distributions. The number-weighted average particle radii were 18 ± 2 and 20 ± 3 nm. The radii obtained from LS measurements were based on the z-averages (of either *R*_G_^2^ for the gyration radius or the diffusion coefficient for the hydrodynamic radius) and thus were larger than those from TEM [55]. Moreover, cryo-TEM showed a PS core surrounded by the collapsed inner layer of the partially quaternized P4VP shell, while the outer part of the shell and PEO corona which were hydrated were most likely not visible because of the low contrast in TEM. The fact that the TEM showed non-swollen structures which were not affected by the pH changes also explains why TEM, unlike LS, showed no significant difference in the micelle size at different pH levels.

### 3.4. Release of Alizarin from SVE-FPBA Micelles Triggered by Diols

**Scheme 3:**
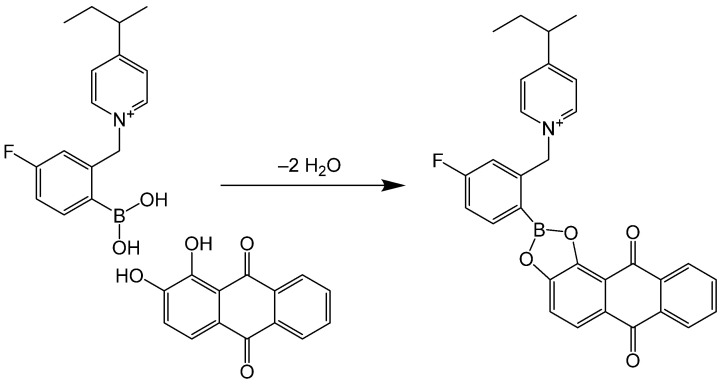
Formation of ester bond between alizarin and FPBA moiety of SVE copolymer.

Alizarin (AL), as a geminal diol, forms an ester bond with FPBA moieties in the SVE-FPBA copolymer (Scheme 3). This reaction is accompanied by a blue shift in the AL absorption spectrum, which allows for monitoring of AL binding into and releasing from SVE-FPBA micelles using UV-Vis absorption spectroscopy.

The polymer concentration in the stock solution of SVE-FPBA micelles loaded with AL was 1.85 mg/mL, corresponding to a molar concentration of FPBA moieties *c*_FPBA_ = 2.2 mM. The micelles contained an amount of AL equimolar to FPBA. For the release measurements, the solution was diluted to a polymer concentration of 1 μg/mL to achieve an optimal AL concentration for spectrophotometric detection of the release. Such a low concentration of SVE-FPBA micelles could also be regarded as safe with respect to their cytotoxicity. (It was proven that block copolymer micelles with PEO coronas were not cytotoxic at mass concentrations about 0.1 mg/mL [48]).

**Figure 5:**
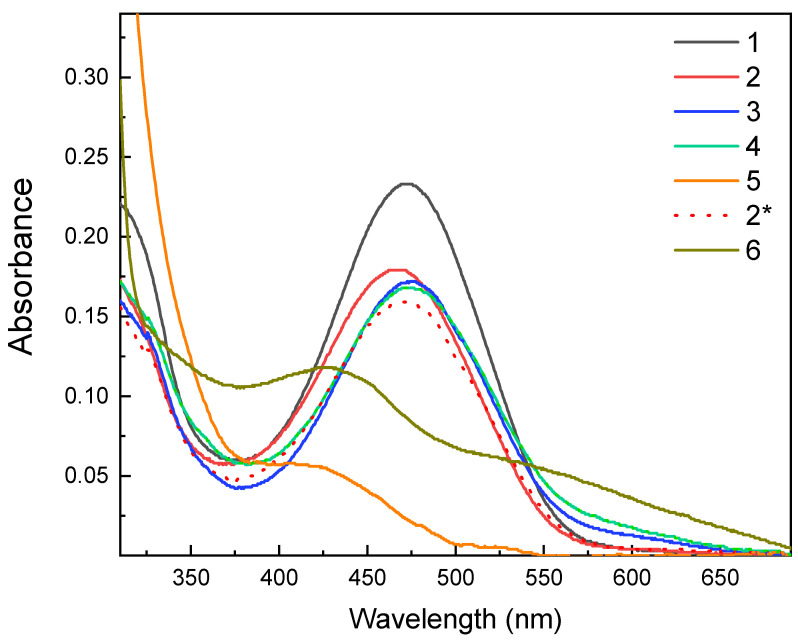
UV-Vis absorption spectra of AL embedded in SVE-FPBA micelles (curve 1) and changes in the spectrum immediately after addition of HCl (curve 2), galactose (curve 3), fructose (curve 4) and ascorbic acid (curve 5). Curve 2* shows the spectrum 72 h after addition of HCl. Curve 6 shows UV-Vis absorption spectrum of AL in aqueous solution of ascorbic acid.

At first, the response of the ester bonds of AL in AL-loaded SVE-FPBA micelles to acidic conditions was examined. Figure 5 shows the UV-Vis absorption spectra of AL in SVE-FPBA micelles at pH 7.69 (curve 1) and after adjustment of the pH level to 2.69 by HCl (Curve 2). The absorption maximum almost did not change even after 72 h, indicating that acid hydrolysis of the AL-FPBA ester did not occur (curve 2*).

The same holds for the addition of galactose (curve 3) and fructose (curve 4) to AL-loaded SVE-FPBA micelles in 10-fold stoichiometric excess to AL (the spectra after 72 h overlapped with that for HCl after 72 h). This means that neither of the two saccharides was capable of a transesterification reaction at the FPBA moieties in SVE-FPBA However, the addition of ascorbic acid (curve 5) in 10-fold stoichiometric excess to AL triggered the release of AL from the micelles, as seen on the spectrum, the maximum of which corresponded to free AL in an acidic aqueous solution. (The UV-Vis spectrum of free AL in the aqueous solution of ascorbic acid is shown for comparison as curve 6.)

It should be pointed out that even though the addition of ascorbic acid decreased the pH of the solution to ca. 2.5, the release was not caused by acid hydrolysis of the AL-FPBA ester, as proven by the experiment with the addition of HCl. The difference in the reactivity of ascorbic acid compared with galactose and fructose was rather related to the vicinal diol group of ascorbic acid with OH groups at sp^2^-hybridized carbons. Such geminal diols are generally more reactive with phenylboronic acid compared with sp^3^ geminal diols because of higher acidity and more favorable conformation [56].

## 4. Conclusions

The stimuli-responsive PS-*b*-P4VP-*b*-PEO (SVE) triblock terpolymer was functionalized by post-polymerization modification with 2-bromomethyl-4-fluorophenylboronic acid (FPBA) in *N,N*-dimethylformamide (DMF), yielding the SVE-FPBA copolymer. The quaternization of the nitrogen atoms in P4VP units increased the solubility of the polymer at higher pH values. The ^1^H NMR spectra of the functionalized triblock confirmed the presence of both quaternized and non-quaternized P4VP units, which were used to determine the degree of quaternization.

Stable SVE-FPBA micelles were prepared by the transfer of SVE-FPBA solutions in methanol into water by dialysis. Light scattering measurements showed solvent swelling of the P4VP shell in micelles transferred to acidic solutions with a pH level near five. Lowering the pH below approximately four did not further change the morphology of the micelles.

The SVE-FPBA micelles encapsulated alizarin (AL), which formed ester bonds with the FPBA moieties. While saccharides such as fructose and galactose were not able to trigger the release of AL from the SVE-FPBA micelles, and the system was responsive to ascorbic acid, whose sp^2^ hybridized vicinal diol group underwent a transesterification reaction, with AL-FPBA triggering the release of AL from the micelles. Therefore, considering the stability of the boron-diol ester, we proved that systems based on the boron-diol chemistry can be promising for targeted drug delivery requiring a long drug circulation time in the body and an efficient carrier for contrast agents.

## Figures and Tables

**Table 1:** Light scattering data (mean ± s.d. of three measurements). *R*_G_ =radius of gyration, *M*_W_ = weight-averaged molecular mass, *R*_H_ = hydrodynamic radius, *σ* = width of the distribution function of diffusion coefficients and *ζ* = zeta potential.

Sample	*R*_G_ (nm)	*M*_W_ × 10^−6^ (g/mol)	*σ*	*R*_H_ (nm)	*R*_G_/*R*_H_	*ζ* (mV)
A-SVE-FPBA pH 5.10	30 ± 2	3.4 ± 0.1	0.15 ± 0.04	44 ± 1	0.68	40 ± 6
A-SVE-FPBA pH 2.50	–	–	–	–	–	20 ± 2
N-SVE-FPBA pH 7.20	18 ± 3	4.5 ± 0.2	0.18 ± 0.04	29 ± 2	0.62	40 ± 1

## Data Availability

Not applicable.

## Abbreviations

AL = alizarin, FPBA = 2-bromomethyl-4-fluorophenylboronic acid, PBA = phenylboronic acid, P4VP = poly(4-vinylpyridine), P4VP-FPBA = poly(4-vinylpyridine) quaternized with 2-bromomethyl-4-fluorophenylboronic acid, Pyr-FPBA = N-[(5-fluoro-2-boronatophenyl)methyl] pyridinium bromide)], SVE = poly(styrene)-b-poly(4-vinyl pyridine)-b-poly(ethylene oxide), and SVE-FPBA = poly(styrene)-b-poly(4-vinyl pyridine)-b-poly(ethylene oxide) quaternized with 2-bromomethyl-4-fluorophenylboronic acid
